# The Converging Roles of DNA Damage Resolution and Redox Dysregulation in Plasma Cell Transformation During the Evolution of Multiple Myeloma

**DOI:** 10.3390/ijms27198595

**Published:** 2026-09-25

**Authors:** Panagiotis Malamos, Konstantinos Koutoulogenis, Maria Gavriatopoulou, Evangelos Terpos, Vassilis L. Souliotis

**Affiliations:** 1 Institute of Chemical Biology, National Hellenic Research Foundation, 116 35 Athens, Greece; pmalamos@eie.gr; 2 Department of Nutrition and Dietetics, School of Health Science and Education, Harokopio University, 176 76 Athens, Greece; konkout@hua.gr; 3 Department of Clinical Therapeutics, School of Medicine, National and Kapodistrian University of Athens, 115 28 Athens, Greece; mgavria@med.uoa.gr (M.G.); eterpos@med.uoa.gr (E.T.)

**Keywords:** multiple myeloma (MM), myelomagenesis, bone marrow plasma cells (BMPCs), oxidative stress, DNA damage response (DDR), nucleotide excision repair (NER), double-strand break repair, chromatin condensation, apoptotic sensitivity

## Abstract

Increasing evidence indicates that dysregulated DNA damage response (DDR) pathways and persistent oxidative stress contribute to genomic instability, increase mutational burden, and promote disease progression. This study investigated the interplay between DDR-related parameters and redox status throughout Multiple Myeloma (MM) evolution. Bone marrow plasma cells (BMPCs) from 16 patients with monoclonal gammopathy of undetermined significance (MGUS), 18 with smoldering multiple myeloma (SMM) and 15 with MM, were analyzed. Measurements included redox status, assessed by the reduced-to-oxidized glutathione (GSH-to-GSSG) ratio, abasic sites, and DDR-related parameters. Baseline DNA damage, abasic site burden, and the kinetics of UVC-induced DNA damage resolution and melphalan-induced γH2AX signal removal were progressively altered across disease stages. Moreover, disease progression was associated with a gradual decrease in chromatin condensation, apoptotic sensitivity, and the GSH-to-GSSG ratio. Multivariate statistical analyses identified distinct DDR-associated patterns that effectively discriminated among patients at different disease stages. Together, the transition from MGUS to SMM and ultimately to MM is characterized by progressive alterations in DNA damage processing, DDR-related signaling, chromatin organization, and redox status. A deeper understanding of these interconnected processes may yield valuable insights into disease progression, improve risk stratification, and guide the development of targeted therapeutic strategies for MM.

## 1. Introduction

Hematologic carcinogenesis is a multistep process through which normal hematopoietic stem or progenitor cells transform into malignant clones, ultimately leading to hematologic malignancies such as leukemia, lymphoma, or multiple myeloma (MM) [1,2]. This process is driven by the accumulation of genetic and epigenetic alterations that disrupt normal blood cell differentiation, proliferation, and survival. MM is a plasma cell dyscrasia characterized by the overproduction of monoclonal immunoglobulins (M protein or paraprotein) and is commonly associated with end-organ damage summarized by the acronym CRAB: calcium elevation, renal failure, anemia, and bone lesions [3].

Unlike solid tumors, which are classified according to their tissue of origin and usually metastasize at later stages, MM is generally systemic at diagnosis. Plasma B cells, from which MM originates, are inherently capable of migrating between bone marrow and lymphoid tissues [4]. The malignant transformation of plasma cells appears to involve three major processes: V(D)J recombination in precursor B cells within the bone marrow, errors in somatic hypermutation (SHM) during plasma cell generation in germinal centers, and abnormal class-switch recombination (CSR), which disrupts immunoglobulin heavy-chain expression and promotes migration and persistence of plasma cells in the bone marrow [5].

MM progression follows distinct precursor stages characterized by accumulating genetic abnormalities and increasing plasma cell proliferation [6]. The disease spectrum advances from monoclonal gammopathy of undetermined significance (MGUS) to smoldering multiple myeloma (SMM) and finally to symptomatic MM [7]. MGUS affects approximately 3% of individuals over 50 years old and is defined by <10% clonal plasma cells in the bone marrow and low monoclonal protein levels (<3 g/dL), with an annual progression risk of about 1% [8]. SMM is an intermediate asymptomatic stage characterized by 10–59% clonal plasma cells and/or elevated M protein levels (≥3 g/dL), with a progression risk of approximately 10% annually during the first five years [9]. Active MM is defined by >10% clonal plasma cells accompanied by CRAB features or other myeloma-defining biomarkers [10].

The DNA damage response (DDR) network is a highly conserved signaling system that detects DNA lesions and coordinates repair processes to preserve genomic integrity. DDR functions as a surveillance mechanism by activating repair pathways, inducing cell cycle arrest, or triggering apoptosis to prevent mutation propagation [11]. During carcinogenesis, DDR shifts from a tumor-suppressive mechanism in early stages to an error-prone, tumor-promoting system in advanced disease. This transition involves genetic and epigenetic alterations in DDR-related genes, leading to genomic instability, replication stress, and resistance to cell death [12]. DDR has been strongly implicated in myelomagenesis, acting as a double-edged sword that supports both genomic instability and malignant plasma cell survival. MM cells exhibit constitutive DNA damage, mainly driven by replication stress, and depend on dysregulated DNA repair pathways to avoid apoptosis during progression from MGUS to active MM [13,14].

Redox status, defined as the balance between reactive oxygen species (ROS) and antioxidants, also plays a central role in carcinogenesis. ROS contribute to DNA damage, activate pro-tumorigenic signaling pathways, and facilitate tumor adaptation to metabolic stress [1]. Oxidative stress induces direct DNA damage and activates stress response pathways, including Nrf2 (nuclear factor erythroid 2-related factor 2), a key regulator of antioxidant defense, and NF-κB (nuclear factor kappa B), which regulates inflammatory and pro-survival signaling [15]. Chronic ROS exposure disrupts normal gene expression, impairs cell communication, and enhances proliferation. It also promotes metabolic reprogramming, angiogenesis [16], and epithelial–mesenchymal transition (EMT) [17], thereby facilitating invasion and metastasis. Importantly, ROS exert concentration-dependent effects: moderate levels promote genomic instability, proliferation, angiogenesis, and metastasis, whereas excessive ROS accumulation induces cell cycle arrest and programmed cell death [18,19,20]. In MM, progression from MGUS to SMM and active disease is associated with increasing oxidative stress, driven by expanding plasma cell burden and enhanced immunoglobulin production, alongside the upregulation of antioxidant defenses that contribute to chemoresistance [21].

Together, dysfunctional DDR and oxidative stress are major contributors to MM pathogenesis and progression. However, despite extensive investigation of these mechanisms individually, their interplay during disease-stage transitions remains incompletely understood. In light of these considerations, we tested the hypothesis that progressive MM development is associated with stage-dependent alterations in DDR efficiency and redox balance, which together promote plasma cell survival and disease progression. We therefore investigated the relationships among baseline DNA damage, DNA damage resolution capacity, apoptotic sensitivity, and redox status in malignant plasma cells from patients with MGUS, SMM, and MM.

## 2. Results

### 2.1. Alterations in DDR Parameters and Oxidative Stress During Malignant Transformation

To elucidate the interconnected roles of the DDR network and cellular redox balance during malignant progression, DDR-associated parameters and the GSH-to-GSSG ratio were evaluated in BMPCs isolated from patients with MGUS (*n* = 16), SMM (*n* = 18), and MM (*n* = 15) at baseline.

Initially, untreated BMPCs were analyzed to determine basal levels of DNA damage, including DNA strand breaks and alkali-labile sites, using the alkaline comet assay. In parallel, γH2AX immunofluorescence was assessed as a surrogate marker of double-strand break (DSB)-associated signaling and DDR activation. In addition, abasic sites—well-established markers of DNA damage and cellular oxidative stress—were quantified using an assay that specifically detects the open-ring aldehydic form of abasic lesions.

We found that DNA lesion levels measured by the comet assay were significantly elevated in MM samples compared with both MGUS and SMM samples (Table S1; Figure 1A,B). In parallel, γH2AX foci levels and abasic site accumulation exhibited a progressive and statistically significant increase from MGUS to SMM and ultimately to MM (Table S1; Figure 1C–E), indicating a gradual accumulation of endogenous DNA damage during myelomagenesis. Given that oxidative stress is a major endogenous source of DNA strand breaks, alkali-labile lesions, and abasic sites, the intracellular GSH-to-GSSG ratio, a well-established indicator of cellular redox status, was subsequently evaluated. A significant and progressive decline in this ratio was observed across disease stages, with the lowest values detected in MM samples (Figure 1F).

We next examined whether the progressive accumulation of DNA damage in malignant plasma cells was associated with changes in their capacity to resolve DNA damage. To assess the kinetics of UVC-induced DNA damage resolution, BMPCs were exposed to 5 J/m^2^ UVC irradiation and subsequently cultured in the appropriate medium for up to 6 h. The levels of DNA strand breaks and alkali-labile sites were monitored at different time points following irradiation using the alkaline comet assay. In all patient groups, DNA damage levels reached a maximum at 1 h after irradiation and progressively declined thereafter (Figure 2A). Notably, BMPCs from MGUS patients exhibited significantly higher levels of residual comet-detectable DNA damage following UVC exposure compared with SMM and MM samples, as reflected by the area under the curve (AUC) values (Table S1; Figure 2B). To further characterize the kinetics of UVC-induced DNA damage resolution during the post-irradiation recovery period, the percentage reduction in mean Olive Tail Moment (OTM) was determined. Specifically, UVC-induced DNA damage resolution was expressed as the percentage reduction in the mean OTM from the peak value observed at 1 h after UVC irradiation to the final measurement at 6 h. Statistical analysis revealed a significantly greater reduction in UVC-induced DNA damage in both SMM and MM patients compared with MGUS patients (both *p* < 0.05; Table S2). Although MM patients exhibited a higher mean percentage of UVC-induced DNA damage resolution than SMM patients (56.63 ± 12.67% versus 48.44 ± 6.83%, respectively), this difference did not reach statistical significance.

Chromatin architecture is known to critically influence DNA repair accessibility and efficiency [22]. Therefore, micrococcal nuclease digestion assays were performed in baseline BMPC samples, focusing on the *N-ras* gene locus, to assess disease stage-dependent alterations in local chromatin organization [23]. BMPCs derived from MGUS patients exhibited a relatively compact chromatin configuration, characterized by the presence of higher-order chromatin structures (Figure 2C). In contrast, samples from SMM and MM patients displayed a progressively more relaxed chromatin organization, predominantly consisting of mono-, di-, and tri-nucleosomal fragments. These observations demonstrate a progressive increase in chromatin accessibility at the *N-ras* locus across disease stages, potentially facilitating increased accessibility of DNA repair machinery at this genomic region.

Next, BMPCs were exposed to melphalan (100 μg/mL for 5 min), incubated in drug-free medium for up to 48 h, and γH2AX foci were quantified by confocal microscopy. Maximal γH2AX levels were observed 8 h after treatment, followed by a progressive decline throughout the recovery period (Figure 2D). Importantly, MGUS, SMM, and MM samples exhibited significantly different persistence of melphalan-induced γH2AX foci, with their levels decreasing progressively across disease stages (Table S1, Figure 2E). To further characterize these differences in γH2AX kinetics, the extent of signal reduction during the recovery period was quantified. Specifically, the percentage of γH2AX signal removal was calculated based on the reduction in the mean number of γH2AX foci per nucleus from the peak level observed at 8 h after melphalan treatment to the final measurement at 48 h. Median γH2AX removal increased across disease stages, with values of 55.17 (IQR, 10.78)% for MGUS, 65.54 (IQR, 8.83)% for SMM, and 77.13 (IQR, 14.46)% for MM. Statistical analysis demonstrated significant differences between all groups (all *p* < 0.05) (Table S2).

Finally, apoptotic sensitivity was evaluated 24 h after exposure to increasing concentrations of melphalan (0–150 μg/mL). A progressive increase in the melphalan concentration required to induce apoptosis was observed from MGUS to MM samples, indicating a gradual reduction in apoptotic sensitivity during disease progression (Figure 2F).

Collectively, these findings indicate that progression across MGUS, SMM, and MM disease stages is associated with progressive accumulation of oxidative stress and endogenous DNA damage, accompanied by chromatin relaxation, alterations in UVC-induced DNA damage resolution and γH2AX signal removal, as well as the gradual acquisition of resistance to apoptosis.

### 2.2. Statistical Analysis of DDR Parameters

Statistical analysis of the DDR parameters was also performed. The correlation matrix (Table 1) revealed a complex yet coherent and biologically meaningful pattern of associations among the investigated DDR parameters. Overall, Spearman’s correlation analysis demonstrated that baseline GSH/GSSG, baseline γH2AX foci, baseline AP sites, γH2AX signal removal (AUC), and apoptotic sensitivity were interconnected through statistically significant positive and negative correlations of moderate to strong strength. In contrast, UVC-induced DNA damage resolution (AUC) appeared to have a more independent contribution, showing only weak positive correlations with baseline DNA damage (rho = 0.335, *p* < 0.05) and baseline GSH/GSSG (rho = 0.295, *p* = 0.05). The strongest association was observed between γH2AX signal removal (AUC) and baseline AP sites, demonstrating a strong negative correlation (rho = −0.780, *p* = 0.001).

To further characterize the phenotypic relationships among the study groups based on their integrated DDR parameter profiles, Hierarchical Cluster Analysis was performed. At the highest rescaled distance, the dendrogram (Figure 3) resolved into two principal clusters (C1 and C2), indicating the presence of two biologically distinct groups based on the integrated DDR parameter profile. Cases 1–16 corresponded to the MGUS group, cases 17–34 to the SMM group, and cases 35–49 to the MM group.

The first principal cluster (C1) comprised the majority of MGUS samples together with a subset of SMM cases. This cluster included samples 31, 32, 14, 11, 12, 16, 26, 13, 28, 8, 9, 5, 7, 4, 29, 30, 10, 15, 25, 33, 1, 3, 38, 27 and 22. Within C1, a tightly grouped sub-cluster was identified (B1), consisting predominantly of MGUS cases, together with a limited number of SMM samples and a single MM outlier (case 38). This sub-cluster exhibited a relatively homogeneous DDR parameter profile characterized by preserved DNA damage processing/resolution capacity and antioxidant redox status, consistent with the indolent biological behavior of MGUS. The presence of several SMM cases within this branch further supports the concept of SMM as a biologically intermediate stage between MGUS and symptomatic MM.

The second branch of C1 (B2) was enriched with SMM and MM cases, with only limited representation of MGUS. This branch included samples 2, 17, 18, 39, 45, 44, 47, 19, 23, 6, 21, 20, 24, 34, 46, 48, 42 and 49. Within B2, two clearly defined sub-clusters were identified. The first sub-cluster was dominated by SMM cases (17, 18, 19, 20, 21, 23, 24 and 34), together with a small number of MM cases and only two MGUS outliers (cases 2 and 6), suggesting a transitional molecular phenotype.

The second principal cluster (C2) consisted exclusively of MM cases. These samples clustered tightly together at low rescaled distances, indicating a highly homogeneous and reproducible disease-specific biomarker signature. Furthermore, the close proximity of the remaining MM cases in B2 to C2, together with the presence of transitional SMM cases, supports a gradual biological continuum from SMM to overt MM rather than an abrupt separation between these disease stages.

## 3. Discussion

Alterations in DDR pathways and redox status are recognized hallmarks of carcinogenesis. During tumor development, ROS-induced DNA damage progressively accumulates despite the adaptive activation of DNA repair pathways, thereby promoting genomic instability while enabling the survival of damaged cells rather than their elimination through apoptosis. In the present study, we investigated the interplay between oxidative stress and DDR-related parameters during MM progression.

We found that plasma cells undergoing myelomagenesis exhibit a progressive accumulation of endogenous DNA lesions, including DNA strand breaks, alkali-labile sites, and apurinic/apyrimidinic sites (Figure 1B,D,E) [24]. This increase may result from increased DNA damage formation, defective processing/resolution of DNA lesions, or a combination of both processes. To evaluate the contribution of endogenous damage formation, we assessed oxidative stress, a major intrinsic source of DNA lesions. Progression from MGUS to SMM and active MM was associated with a gradual decline in the GSH/GSSG ratio (Figure 1F), indicating a shift toward a more oxidizing intracellular environment. These findings are consistent with previous studies demonstrating that disruption of redox status is a central feature of cancer progression [25]. Indeed, in MM, malignant plasma cells produce large amounts of monoclonal immunoglobulins, resulting in endoplasmic reticulum stress and excessive ROS generation [26]. This process constitutes both a hallmark of the disease and a potential therapeutic target [21,27]. Previous studies have consistently documented systemic redox imbalance in MM, characterized by diminished antioxidant defenses and increased pro-oxidant activity. Antioxidant molecules and enzymes, including vitamins C and E, glutathione peroxidase, catalase, and superoxide dismutase, are reduced in MM patients [28,29]. Lower paraoxonase and arylesterase activities have also been associated with poor prognosis [30], whereas decreased circulating SCARA3 levels correlate with disease progression [31]. In parallel, biomarkers of oxidative stress, including AOPPs, MDA, NO, AGEs, and 8-isoprostanes, are elevated in MM [30,32]. Because oxidative stress is a major source of AP lesions, the progressive increase in oxidative stress observed in our study may contribute to the accumulation of AP sites during myelomagenesis [33]. AP sites are among the most frequent endogenous DNA lesions and can interfere with replication and transcription, thereby promoting mutagenesis and genomic instability [34].

To investigate whether alterations in DNA damage resolution contribute to the observed accumulation of endogenous DNA damage, we monitored the kinetics of comet-detectable DNA strand breaks and alkali-labile sites following UVC exposure. We observed a progressively faster decline in residual DNA lesions from MGUS to MM (Figure 2A,B), indicating progressively faster resolution of UVC-induced comet-detectable DNA damage. NER is responsible for the removal of DNA lesions induced by ultraviolet radiation, environmental carcinogens, and several chemotherapeutic agents. However, because the alkaline comet assay does not directly quantify UV photoproduct removal, the observed kinetics cannot be considered a direct measure of NER activity and may reflect the integrated processing of UVC-induced DNA lesions. Dysregulation of NER has been implicated in MM progression and treatment response, particularly through altered expression of NER-related genes [35]. Among these, *ERCC3*, which encodes the DNA helicase XPB, is frequently overexpressed and has been associated with adverse clinical outcomes. Direct quantification of UV photoproducts, such as CPDs or 6-4PPs, would be required to determine whether the observed differences are specifically attributable to altered NER activity; however, such measurements were not performed in the present study and additional biological material from the cohort is not available.

Moreover, we observed progressively faster removal of melphalan-induced γH2AX foci from MGUS to MM (Figure 2D,E), reflecting differences in the kinetics of DSB-associated DDR signaling and γH2AX signal removal. As DSBs represent one of the most deleterious forms of DNA damage, efficient detection, signaling, and repair of these lesions are critical for preserving genome stability [36,37]. Accumulating evidence indicates that dysregulation of DSB repair contributes substantially to MM pathogenesis and treatment resistance. Multiple components of the DSB repair network are aberrantly expressed or functionally altered in MM. For example, MM cells overexpress several DNA repair genes, including *RAD50* and *RAD51* [38,39], while elevated expression of *PRKDC*, *PARP1*, and *BRCA1* has been associated with unfavorable prognosis following high-dose melphalan and autologous stem cell transplantation [40]. In addition, polymorphisms in DNA repair genes such as *RAD51*, *MUTYH*, *PCNA*, *PARP1*, *TPMT*, and *OGG1* have been linked to MM susceptibility and disease progression [41].

Collectively, these findings support the notion that altered DNA damage processing and resolution may represent an important adaptive feature during myeloma evolution. Although the faster resolution of UVC-induced comet-detectable DNA damage and the more rapid removal of melphalan-induced γH2AX foci are consistent with altered DNA damage processing and/or repair, these functional assays do not distinguish between enhanced repair activity and other processes that may influence the kinetics of damage resolution. Importantly, the alkaline comet assay does not directly measure the removal of UV photoproducts, while γH2AX foci reflect both DSB-associated signaling and its resolution and therefore cannot be equated directly with completion of DSB repair. The observed kinetics may also be influenced by differences in baseline DNA damage burden, lesion recognition and processing, cell-cycle or proliferation status, and selective loss of severely damaged cells during ex vivo manipulation. Similarly, differences in cellular viability following genotoxic exposure could contribute to the observed reduction in residual damage. Although these factors cannot be completely excluded by the present experimental design, the concordant patterns observed following two distinct genotoxic challenges support the presence of stage-associated alterations in DNA damage processing and resolution. Future studies incorporating direct pathway-specific repair assays, including direct quantification of UV photoproducts, together with cell-cycle normalization and detailed viability analyses, will be required to determine the relative contribution of individual mechanisms.

Chromatin organization is a major determinant of DNA repair efficiency [42,43]. We observed a progressive increase in chromatin accessibility at the *N-ras* locus during disease progression (Figure 2C), consistent with previous reports showing that advanced tumor stages are characterized by chromatin decompaction and enhanced transcriptional plasticity [44]. Chromatin remodeling, DNA methylation, and histone modifications collectively regulate gene expression, DNA repair, and drug responsiveness [23,45]. MM is characterized by extensive dysregulation of histone modifications and chromatin-remodeling complexes that promote pro-survival chromatin states [23,46,47]. For example, increased nuclear heparanase expression has been shown to drive transcriptional programs associated with aggressive disease behavior and chromatin relaxation [48]. Enhanced chromatin accessibility may therefore facilitate the recruitment of DNA repair factors to damaged sites, contributing to the more efficient processing of DNA lesions observed in advanced disease stages. Interestingly, the progressive increase in chromatin accessibility observed at the *N-ras* locus may be compatible with a more permissive local chromatin environment for DNA damage processing. However, because chromatin organization was assessed at a single genomic locus, these findings cannot be extrapolated to genome-wide chromatin relaxation. Whether similar alterations occur at other genomic regions during myeloma progression remains to be determined using genome-wide chromatin accessibility or chromatin profiling approaches.

Finally, progression from MGUS to SMM and overt MM was accompanied by a gradual reduction in apoptotic sensitivity (Figure 2F), consistent with previous studies reporting progressive impairment of apoptosis during disease evolution [49]. MM cells acquire multiple mechanisms that suppress both intrinsic and extrinsic apoptotic pathways [50]. Overexpression of anti-apoptotic members of the BCL-2 family, particularly MCL1 and BCL-XL, together with reduced expression or activity of pro-apoptotic mediators such as BIM and BAX, promotes malignant plasma cell survival [51,52]. In addition, genomic instability and the progressive accumulation of secondary genetic alterations contribute to the selection and expansion of apoptosis-resistant clones. Concurrently, malignant plasma cells become increasingly reliant on adaptive stress-response mechanisms, including autophagy and the unfolded protein response (UPR), to cope with the proteotoxic stress generated by sustained immunoglobulin production [53]. Furthermore, interactions with the bone marrow microenvironment reinforce anti-apoptotic signaling through activation of the IL-6, NF-κB, and PI3K/AKT pathways, thereby enhancing proliferation, immune evasion, and resistance to therapy [54,55].

Our hierarchical clustering analysis (HCA) further supports the concept that progressive alterations in DDR pathways accompany plasma cell transformation during myelomagenesis (Figure 3). Using an integrated panel of DDR-related parameters, HCA achieved a partial but biologically meaningful segregation of MGUS, SMM, and MM cases. Most MGUS samples clustered together, whereas the majority of MM samples formed a distinct and highly homogeneous cluster, consistent with the progressive acquisition of a stage-associated DDR phenotype during malignant evolution. In contrast, SMM cases were distributed between the two principal clusters, reinforcing the notion that SMM represents a biologically heterogeneous and transitional stage bridging MGUS and symptomatic MM. This observation is in agreement with recent models proposing that SMM encompasses lesions with markedly different molecular trajectories and risks of progression [56].

Importantly, the presence of cross-cluster cases, including an MM case clustering with predominantly MGUS samples and isolated MGUS cases clustering with advanced disease, highlights the biological overlap between clinical stages and suggests that conventional disease classification may not fully capture the underlying molecular heterogeneity. These findings support the potential utility of biomarker-driven multivariate approaches as complementary tools for refining biological stratification beyond conventional clinical staging [57]. Although exploratory and limited by sample size, the observed clustering pattern is consistent with a gradual continuum of DDR remodeling during disease progression rather than discrete transitions between MGUS, SMM, and MM. Taken together, these findings indicate that alterations in oxidative stress, DNA damage accumulation, DNA damage processing and resolution, and apoptotic responsiveness are collectively associated with the biological differences observed across MGUS, SMM, and MM.

The present study has several limitations, including the relatively modest sample size and the lack of demographic and clinical matching between disease-stage groups. Although clinical characteristics are provided descriptively in Table 2, formal statistical comparisons of these characteristics were not performed, and no multivariable adjustment or sensitivity analysis was undertaken. Therefore, the potential contribution of age or other clinical variables to the observed stage-associated differences cannot be fully excluded. Larger, well-characterized cohorts with appropriate adjustment for potential confounders will be required to determine whether the observed alterations are independently associated with disease stage.

## 4. Materials and Methods

### 4.1. Patients

Forty-nine patients were included: 16 with MGUS (7F/9M; median age 63.5 years, range 38–85), 18 with SMM (6F/12M; median age 66.5 years, range 45–81), and 15 with MM (7F/8M; median age 70.0 years, range 39–88). Clinical characteristics are summarized in Table 2. Diagnoses were made in accordance with the criteria established by the International Myeloma Working Group [58]. The study was approved by the Institutional Review Board and conducted in accordance with the Declaration of Helsinki. All participants provided written informed consent.

### 4.2. Isolation and Preparation of Bone Marrow Plasma Cells

Bone marrow mononuclear cells were isolated from heparinized bone marrow samples by Ficoll–Hypaque density-gradient centrifugation, following standard density-gradient separation procedures and as previously described [59]. Briefly, bone marrow samples were diluted with RPMI-1640 medium and carefully layered onto Ficoll–Hypaque density-gradient medium. Following centrifugation, the mononuclear cell fraction was collected from the interface between the plasma and Ficoll layers and subsequently washed with RPMI-1640 medium. CD138^+^ plasma cells were then enriched from the bone marrow mononuclear cell fraction by positive magnetic selection using anti-CD138 microbeads (Miltenyi Biotec, Bergisch Gladbach, Germany), according to the manufacturer’s instructions. Following isolation and enrichment, cells were cryopreserved in 90% fetal bovine serum (FBS) and 10% dimethyl sulfoxide (DMSO) at −80 °C for up to 1 month prior to analysis. All samples were subjected to identical cryopreservation, storage, thawing, and analytical procedures and were processed and analyzed in a blinded manner. For all assays, BMPCs were cultured in RPMI-1640 supplemented with 10% FBS, penicillin (100 U/mL), streptomycin (100 μg/mL), and L-glutamine (2 mmol/L).

### 4.3. Alkaline Comet Assay

The alkaline comet assay was performed as described in [60]. Briefly, 1 × 10^4^ cells were suspended in PBS (phosphate-buffered saline, pH 7.4) containing 1% low-melting-point (LMP) agarose and subsequently transferred onto glass slides. The slides were then allowed to solidify at 4 °C for 30 min. Following solidification, the slides were immersed in an alkaline lysis solution consisting of 0.01 M Tris (pH 10), 2.5 M NaCl, 0.1 M EDTA, and 1% Triton X-100 and incubated at 4 °C for 2 h. Electrophoresis was subsequently performed for 30 min at 25 V and 225 mA. DNA was stained with SYBR Gold Nucleic Acid Gel Stain (Thermo Fisher Scientific, Waltham, MA, USA; #S11494) and visualized by a Zeiss Axiophot fluorescence microscopy system (Zeiss, Oberkochen, Germany). DNA damage was quantified as Olive Tail Moment by CometScore free software v1.5 (TriTek Corp, Sumerduck, VA, USA), scoring ≥ 200 cells per condition.

### 4.4. Measurement of Ultraviolet C (UVC)-Induced DNA Damage Resolution

Cells were seeded into six-well plates at a density of 1 × 10^6^ cells/mL in PBS. Prior to UVC exposure, each well was covered with 0.5 mL of PBS, corresponding to an approximate liquid depth of 0.53 mm. The cells were subsequently irradiated with UVC light at 254 nm using a Philips 6 W germicidal lamp (Attica, Greece). Irradiation was performed at a dose rate of 0.83 J/m^2^/s for 6 s, resulting in a total UVC dose of 5 J/m^2^. After UVC irradiation, BMPCs were incubated for 0–6 h at 37 °C in the appropriate culture medium, harvested at the indicated time points, and analyzed using the alkaline comet assay [60]. The assay was used to monitor changes in comet-detectable DNA damage throughout the post-irradiation recovery period. DNA damage resolution was quantified based on the reduction in the mean OTM (arbitrary units) from the maximum value observed at 1 h after irradiation to the value measured at 6 h. The percentage of UVC-induced DNA damage resolution was calculated as the percentage reduction in mean OTM between these two time points. This measurement reflects the decrease in detectable DNA damage over the recovery period and does not by itself identify the specific molecular pathway(s) responsible for this process.

### 4.5. Immunofluorescence Quantification of γH2AX Foci

Cells (5 × 10^5^) were seeded on poly-L-lysine-coated coverslips, fixed in 4% paraformaldehyde, and permeabilized with Triton X-100. After blocking, samples were incubated with anti-γH2AX antibody (Cell signaling, Danvers, MA, USA; #80312; 1:400) followed by Alexa Fluor 488-conjugated secondary antibody (Invitrogen, Carlsbad, CA, USA; #481679; 1:1000). Coverslips were mounted with DAPI-containing medium (EverBrite™ Hardset, Biotium, Fremont, CA, USA; #23004) and imaged using a Leica TCS SP-1 confocal microscope (Leica Microsystems, CMS GmbH, Mannheim, Germany). The γH2AX foci were manually scored in 100 nuclei per condition and expressed as the mean number of foci per nucleus. Baseline γH2AX foci were assessed in untreated BMPCs, while following melphalan treatment, γH2AX foci were quantified at the indicated recovery time points. The percentage of γH2AX signal removal was calculated as the percentage reduction in the mean number of γH2AX foci per nucleus from the peak level observed at 8 h after melphalan treatment to the final measurement at 48 h. Data represent the mean of three independent experiments.

### 4.6. Chromatin Accessibility Assay

Chromatin accessibility was assessed by micrococcal nuclease digestion. Cells were incubated in hypotonic buffer, lysed with Nonidet P-40, and nuclei were isolated by centrifugation. Nuclei were digested with micrococcal nuclease (1 U, 5 min, 37 °C; Takara Bio, San Jose, CA, USA; #2910A) in digestion buffer (100 mM Tris–HCl, pH 8.0, 3 mM MgCl_2_, 1 mM CaCl_2_, 50 mM NaCl), and reactions were terminated with proteinase K-containing stop buffer (200 mM Tris–HCl, pH 8.0, 20 mM EDTA, 2% SDS, 200 mM NaCl). Genomic DNA was purified, resolved on 1.5% agarose gels, transferred to nitrocellulose membranes, and hybridized with *N-ras*-specific probe [42].

### 4.7. GSH-to-GSSG and Abasic (AP; Apurinic/Apyrimidinic) Site Quantification

Intracellular redox status was assessed using the GSH/GSSG-Glo assay (Promega, Madison, WI, USA; V6612). The cellular glutathione redox state was evaluated by determining the ratio between reduced glutathione (GSH) and its oxidized form (GSSG) using the GSH/GSSG-Glo Assay (Promega, Madison, WI, USA; V6612), according to the protocol provided by the manufacturer. Briefly, 1 × 10^4^ cells were seeded into a 96-well tissue-culture microplate (Corning Costar, Tewksbury, MA, USA) compatible with luminescence-based measurements. Following cell seeding, 50 μL of Luciferin Generation Reagent was added to each well. The plate was briefly shaken to ensure adequate mixing of the reagent with the samples and was subsequently incubated at room temperature for 30 min. Next, 100 μL of Luciferin Detection Reagent was added to each well, followed by a further incubation period of 15 min at room temperature. The resulting luminescence was then quantified using a SpectraMax M3 microplate reader (Molecular Devices LLC, San Jose, CA, USA). The measured luminescence values were used to determine the relative levels of GSH and GSSG and, consequently, the GSH/GSSG ratio [23].

The extent of DNA damage associated with abasic (AP) sites was assessed using the OxiSelect Oxidative DNA Damage Quantitation Kit (Cell Biolabs, San Diego, CA, USA; STA-324), in accordance with the protocol provided by the manufacturer. The assay is based on the selective derivatization of AP sites through their aldehyde functional groups. Specifically, the Aldehyde Reactive Probe (ARP) recognizes and reacts with the aldehyde moiety that is exposed when the deoxyribose residue at an abasic site adopts its open-chain form. Following this reaction, the AP sites are subsequently biotinylated, allowing their detection through binding to a streptavidin-conjugated enzyme. The intensity of the resulting signal is proportional to the amount of AP-site-containing DNA present in the sample. Quantification was performed by measuring the absorbance of the DNA samples and comparing the obtained values with a standard calibration curve generated using the DNA standard supplied with the kit, which contains a defined number of abasic sites. The concentration of abasic lesions in the experimental samples was subsequently calculated based on their absorbance relative to the corresponding standard curve.

### 4.8. Measurement of Apoptotic Sensitivity

Cells (2 × 10^4^) were treated with melphalan (0–150 μg/mL for 5 min) and then incubated for 24 h in drug-free medium. Apoptotic sensitivity was quantified using the Cell Death Detection ELISA-PLUS kit (Roche Diagnostics, Indianapolis, IN, USA; #11774425001), following the manufacturer‘s protocol. Briefly, cytosolic nucleosomes were captured using anti-histone antibodies and detected with peroxidase-conjugated anti-DNA antibodies. Absorbance was measured photometrically, and the assay determined the enrichment factor (EF) = (absorbance of the treated cells)/(absorbance of the untreated cells). Apoptotic sensitivity was expressed as the drug dose required to induce a specific enrichment factor (EF = 3).

### 4.9. Statistical Analysis

Normality was assessed using the Shapiro–Wilk test. Differences among groups for normally distributed variables were evaluated using one-way analysis of variance (ANOVA), followed by Bonferroni-adjusted post hoc comparisons. For non-normally distributed variables, differences were assessed using the Kruskal–Wallis test, followed by pairwise comparisons with Bonferroni correction. Normally distributed continuous variables are presented as mean ± standard deviation (SD), whereas non-normally distributed variables are presented as median and interquartile range (IQR). Correlations between variables were examined using Spearman’s rank correlation coefficient. Hierarchical Cluster Analysis was performed on DDR parameters using Ward’s linkage method with squared Euclidean distances. The rescaled cluster distances are presented on the *y*-axis, and the observed cases are displayed on the *x*-axis. Statistical analyses were performed using the Statistical Package for the Social Sciences (SPSS for Windows, version 30.0; SPSS Inc., Chicago, IL, USA). A two-sided *p* value < 0.05 was considered statistically significant.

## 5. Conclusions

Our findings highlight a dynamic interplay between oxidative stress and the DDR network during progression from MGUS to MM (Figure 4). The progressive accumulation of endogenous DNA lesions, together with the decline in the GSH/GSSG ratio, is consistent with an increasing burden of oxidative stress during disease evolution. Despite elevated levels of DNA damage, BMPCs exhibit more rapid resolution of experimentally induced, comet-detectable DNA damage following UVC exposure, as well as faster removal of melphalan-induced γH2AX foci. These findings are accompanied by increased chromatin accessibility at the *N-ras* locus, which may reflect a more permissive local chromatin environment for DNA damage processing, and by a gradual reduction in apoptotic sensitivity, consistent with increased tolerance to genotoxic stress. Collectively, these observations support an association between disease progression and coordinated changes in redox homeostasis, DNA damage accumulation and resolution, chromatin organization, and apoptotic sensitivity. Importantly, the observed kinetics of damage resolution represent integrated cellular responses and should not be interpreted as direct evidence of increased intrinsic DNA repair capacity. Although these findings are consistent with progressive alterations in these cellular processes across MGUS, SMM, and MM, the present study does not establish direct causal relationships among them. Further studies incorporating pathway-specific and genome-wide approaches will be required to determine their mechanistic contribution to myeloma evolution.

## Figures and Tables

**Figure 1:**
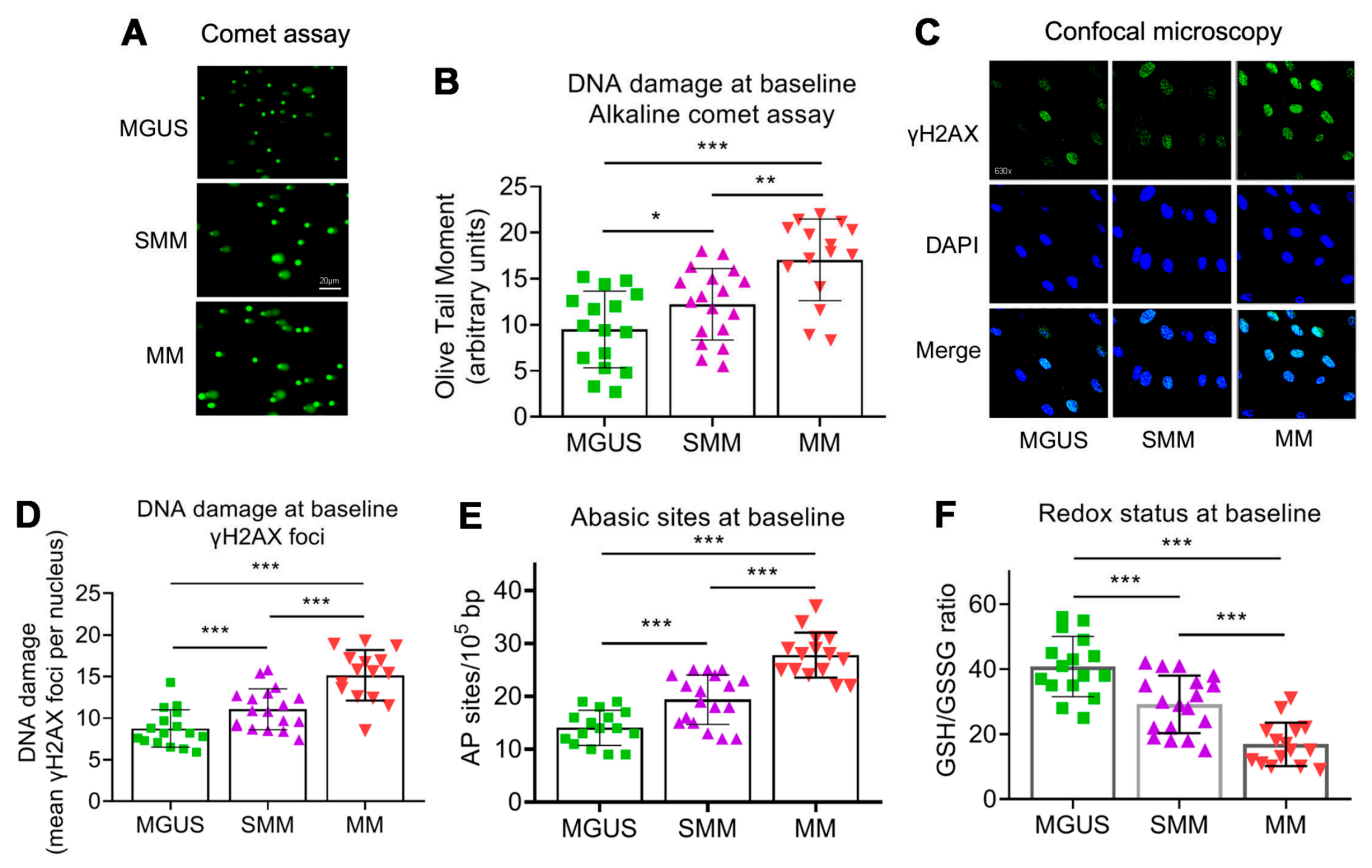
Baseline DNA damage and GSH-to-GSSG ratio in BMPCs. (**A**) Alkaline comet assay images of untreated BMPCs from MGUS, SMM and MM patients; scale bar: 20 μm. (**B**) DNA damage measured by comet assay. (**C**) Representative images from confocal microscopy; upper images, γH2AX staining; middle, cell nuclei labeled with DAPI; bottom, merged images; magnification ×630. (**D**) Measurement of γH2AX foci by confocal microscopy. (**E**) Abasic lesions detected via the streptavidin–biotin interaction. (**F**) The GSH-to-GSSG ratio was also measured. Error bars represent standard deviation (SD); * *p* < 0.05, ** *p* < 0.01, *** *p* < 0.001.

**Figure 2:**
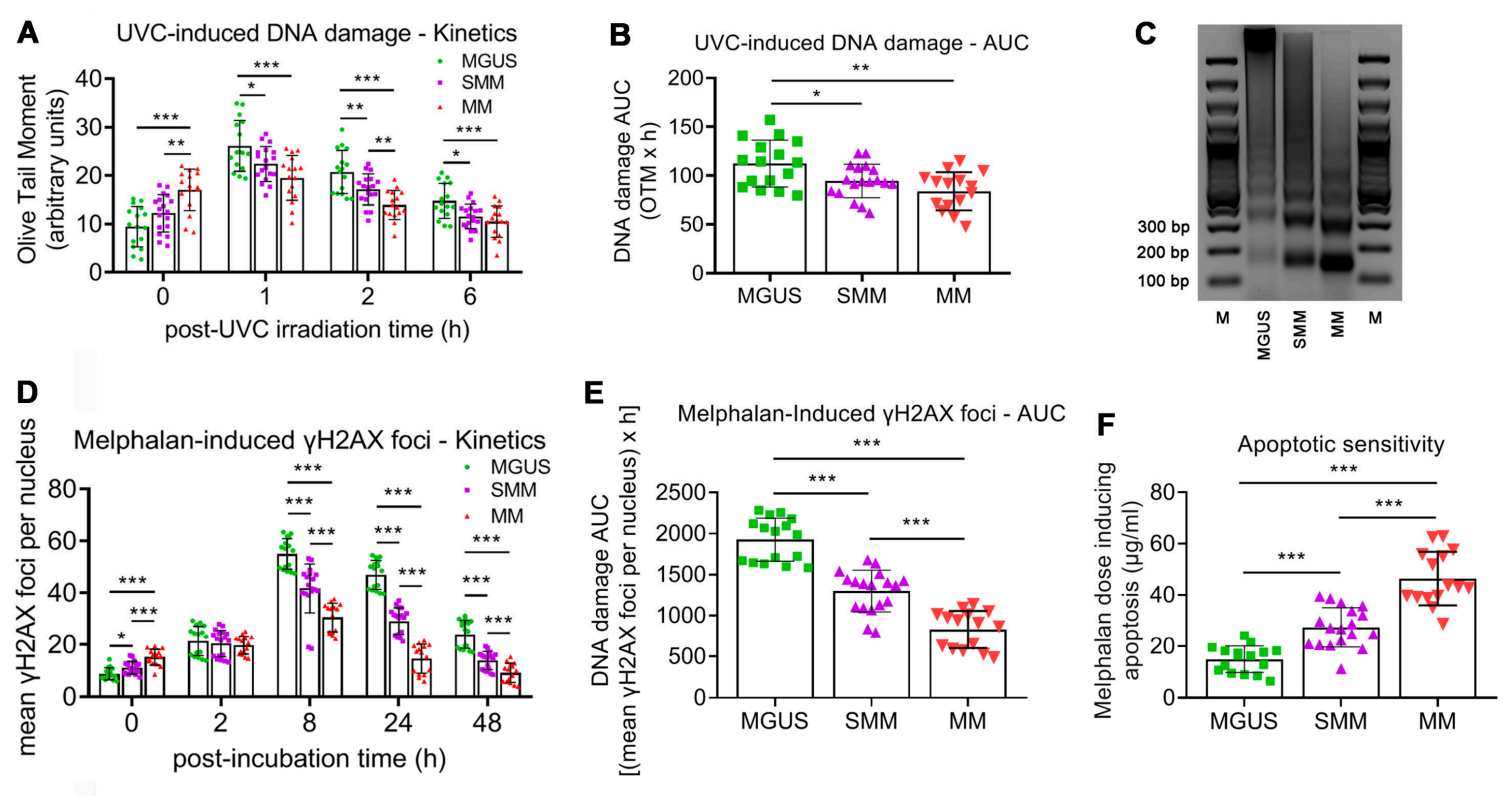
DNA damage resolution and apoptotic sensitivity. (**A**) Kinetics of UVC-induced DNA lesions measured by comet assay, and (**B**) DNA damage burden, expressed as AUC. (**C**) Baseline chromatin condensation in BMPCs. (M), 100 bp DNA ladder; untreated, without micrococcal nuclease digestion. (**D**) Melphalan-induced γH2AX formation and removal kinetics, and (**E**) accumulation of γH2AX expressed as AUC. (**F**) Apoptotic sensitivity 24 h following melphalan treatment. Error bars represent SD; * *p* < 0.05, ** *p* < 0.01, *** *p* < 0.001.

**Figure 3:**
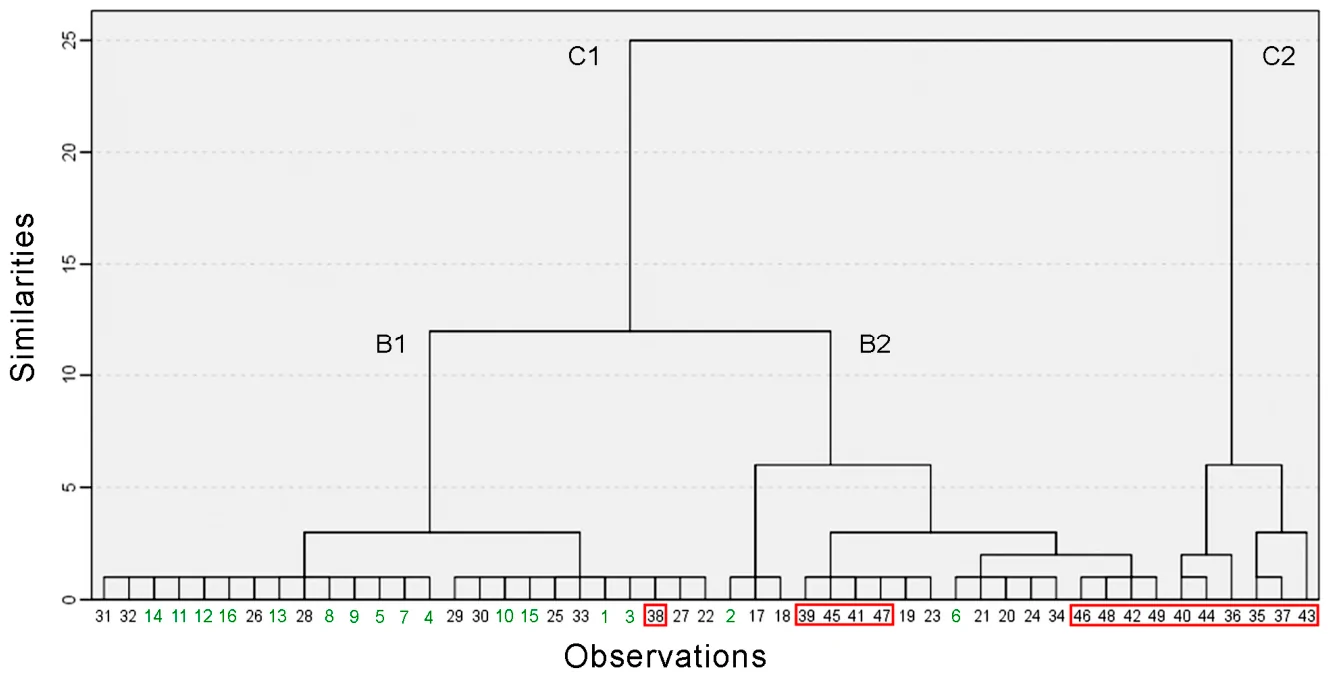
Hierarchical clustering dendrogram of MGUS, SMM, and MM samples based on the measured DDR parameters. Cases 1–16 correspond to the MGUS group (green labels), cases 17–34 to the SMM group (black labels), and cases 35–49 to the MM group (labels enclosed in red rectangles). The vertical axis (*y*-axis) represents the similarity coefficient at which clusters are merged, whereas the horizontal axis (*x*-axis) represents the individual study samples (cases).

**Figure 4:**
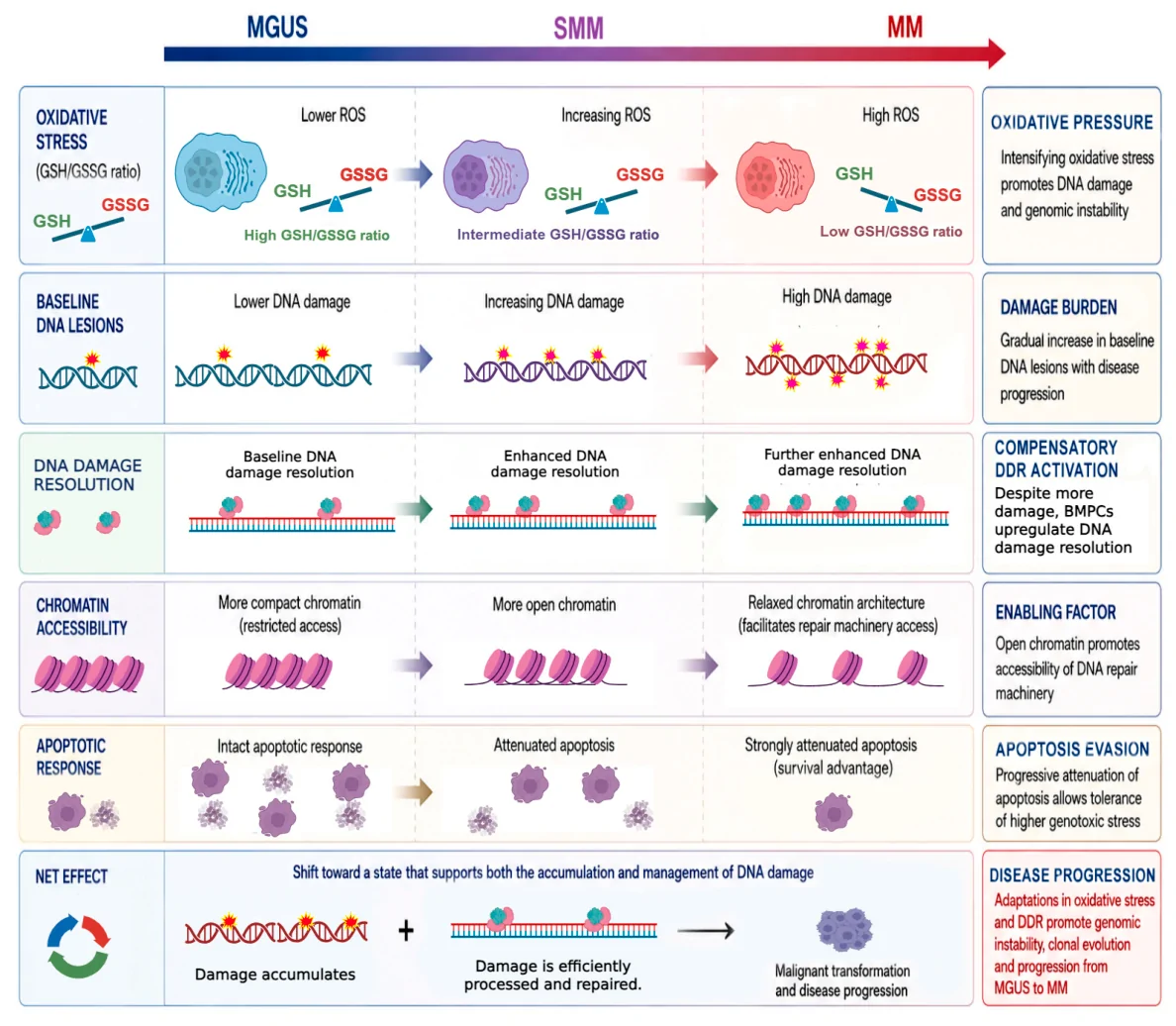
Proposed model of oxidative stress-DDR adaptation during progression from MGUS to MM. Progressive disease is associated with increased oxidative stress and elevated basal DNA damage, accompanied by enhanced DNA damage resolution, increased chromatin accessibility, and reduced apoptotic sensitivity in BMPCs. Figure produced using “BioRender.com (accessed on 1 July 2026)”.

**Table 1:** Spearman’s correlation results between the measured DDR parameters of the investigated groups.

	Baseline DNA Damage	Baseline γH2AX Foci	Baseline GSH/GSSG	Baseline AP Sites	UVC-Induced DNA Damage Resolution (AUC)	γH2AX Signal Removal (AUC)
**Baseline γH2AX foci**	0.480 **					
**Baseline GSH/GSSG**	−0.461 **	−0.517 **				
**Baseline AP Sites**	0.574 **	0.636 **	−0.711 **			
**UVC-induced DNA damage resolution (AUC)**	0.335 *	−0.225	0.295 *	−0.186		
**γH2AX signal removal (AUC)**	−0.480 **	−0.684 **	0.616 **	−0.780 **	0.399 **	
**Apoptotic Sensitivity**	0.498 **	0.674 **	−0.697 **	0.659 **	−0.387 **	−0.762 **

Baseline DNA damage, DNA lesions measured by alkaline comet assay; Apoptotic sensitivity: the lowest concentrations of melphalan required for the induction of apoptosis; * correlation is significant at the 0.05 level (2-tailed); ** correlation is significant at the 0.01 level (2-tailed).

**Table 2:** Patient and disease characteristics.

	Patients		
Characteristic	MGUS (*n* = 16)	SMM (*n* = 18)	MM (*n* = 15)
**Age (years)**			
Median	63.5	66.5	70.0
Range	38–85	45–81	39–88
**Gender**			
Female	7 (43.8%)	6 (33.3%)	7 (46.7%)
Male	9 (56.2%)	12 (66.7%)	8 (53.3%)
**Serum M-protein isotype**			
*Heavy chain*			
IgA	3 (18.8%)	5 (27.8%)	4 (26.7%)
IgG	13 (81.2%)	13 (72.2%)	8 (53.3%)
IgM	-	-	1 (6.7%)
None detected	-	-	2 (13.3%)
*Light chain*			
Kappa light chain	10 (62.5%)	12 (66.7%)	10 (66.7%)
Lambda light chain	6 (37.5%)	6 (33.3%)	4 (26.7%)
None detected	-	-	1 (6.7%)
**ISS stage**			
I	-	-	4 (26.7%)
II	-	-	8 (53.3%)
III	-	-	3 (20.0%)
**Serum β2 microglobulin**			
<3.5 mg/L	14 (87.5%)	14 (77.7%)	6 (40.0%)
3.5–5.4 mg/L	2 (12.5%)	3 (16.7%)	4 (26.7%)
≥5.5 mg/L	-	1 (5.6%)	5 (33.3%)
**Serum albumin**			
<3.5 g/dL	2 (12.5%)	1 (5.6%)	4 (26.7%)
≥3.5 g/dL	14 (87.5%)	17 (94.4%)	11 (73.3%)
**Lactate dehydrogenase**			
Normal (≤225 U/L)	15 (93.8%)	16 (88.8%)	10 (66.7%)
Elevated (>225 U/L)	1 (6.3%)	2 (11.2%)	5 (33.3%)
**Cytogenetic risk**			
Standard	-	-	13 (86.7%)
High	-	-	2 (13.3%)

Ig, immunoglobin; ISS, International Staging System; Cytogenetic risk was defined as high based on the presence of del(17p), translocation t(4;14), and/or translocation t(14;16).

## Data Availability

The original contributions presented in this study are included in the article/Supplementary Materials. Further inquiries can be directed to the corresponding author.

## Supplementary Materials

The following supporting information can be downloaded at https://www.mdpi.com/article/10.3390/ijms27198595/s1.
