# Supplementary material for: The Converging Roles of DNA Damage Resolution and Redox Dysregulation in Plasma Cell Transformation During the Evolution of Multiple Myeloma
Source: Int J Mol Sci. 2026 Sep 25;27(19):8595. doi: 10.3390/ijms27198595 (PMC13664816; doi:10.3390/ijms27198595)
Supplement: Supplementary file 1 [file ijms-27-08595-s001.zip › ijms-4571564-supplementary.pdf]

# Supplementary Materials

## The Converging Roles of DNA Damage Resolution and Redox Dysregulation in Plasma Cell Transformation during the Evolution of Multiple Myeloma

**Malamos P. et al.**

**Table of Contents**

Table S1: Comparisons of the DDR biomarkers across the investigating groups
Table S2. UVC-induced DNA damage resolution (%) and γH2AX signal removal (%) with statistical comparisons among MGUS, SMM, and MM groups

**Table S1:** Comparisons of the DDR parameters across the investigating groups

| | **Diagnosis** | | |
|---|---|---|---|
| | **MGUS ($n$ = 16)** | **SMM ($n$ = 18)** | **MM ($n$ = 15)** |
| | | **Mean (± SD)** | |
| **Baseline DNA damage** | 9.5 (± 4.2) | 12.2 (± 3.9) [a] | 17.1 (± 4.4) [a, b] |
| **Baseline γH2AX foci** | 8.8 (± 2.3) | 11.1 (± 2.4) [a] | 15.2 (± 3.0) [a, b] |
| **Baseline GSH/GSSG** | 40.8 (± 9.3) | 29.2 (± 8.8) [a] | 16.9 (± 6.7) [a, b] |
| **Baseline AP Sites** | 14.1 (± 3.3) | 19.4 (± 4.7) [a] | 27.8 (± 4.2) [a, b] |
| **UVC-induced DNA damage resolution (AUC)** | 112.4 (± 23.9) | 94.6 (± 17.2) [a] | 84.0 (± 19.6) [a] |
| **γH2AX signal removal (AUC)** | 1926.1 (± 263.2) | 1298.9 (± 253.5) [a] | 829.3 (± 228.2) [a, b] |
| | | **Median (IQR)** | |
| **Apoptotic sensitivity** | 16.3 (8.1) | 26.9 (12.9) [a] | 43.1 (16.7) [a, b] |

Significant comparisons: [a] compared with MGUS; [b] compared with SMM; statistical significance for all results was $p < 0.05$;
One-way ANOVA with Bonferroni correction (post hoc analysis) was used for Baseline DNA damage, Baseline γH2AX foci, Baseline GSH/GSSG, Baseline AP Sites and UVC-induced DNA damage resolution (AUC);
Kruskal-Wallis with pairwise comparisons and Bonferroni correction was used for Apoptotic sensitivity;
IQR: Interquartile range, SD: Standard deviation;
Baseline DNA damage: OTM (arbitrary units); Apoptotic sensitivity: melphalan dose inducing apoptosis (μg/ml); Baseline γH2AX: mean γH2AX foci per nucleus; Baseline AP sites: AP sites/$10^5$ bp; UVC-induced DNA damage resolution (AUC): OTM x h; γH2AX signal removal (AUC): (mean γH2AX foci per nucleus) x h

**Table S2.** UVC-induced DNA damage resolution (%) and γH2AX signal removal (%) with statistical comparisons among MGUS, SMM, and MM groups

| DDR marker | MGUS | SMM | MM |
|---|---|---|---|
| | | **Mean (± SD)** | |
| **UVC-induced DNA damage resolution (%)** [a, d] | 35.39 (± 9.88) | 48.44 (± 6.83) [1] | 56.63 (± 12.67) [1] |
| | | **Median (IQR)** | |
| **γH2AX signal removal (%)** [b, c] | 55.17 (10.78) | 65.54 (8.83) [1] | 77.13 (14.46) [1,2] |

[a] UVC-induced DNA damage resolution (%) was calculated as the percentage reduction in the mean number of Olive Tail Moment (arbitrary units) from the peak level observed at 1h after irradiation to the final measurement at 6h;

[b] γH2AX signal removal (%) was calculated as the percentage reduction in the mean number of γH2AX foci per nucleus from the peak level observed at 8h after treatment to the final measurement at 48h;

[c] Kruskal-Wallis;

[d] independent one-way ANOVA;

Significant comparisons: [1] compared with MGUS; [2] compared with SMM; statistical significance for all results was $p < 0.05$
